# Cardiometabolic Index is associated with heart failure: a cross-sectional study based on NHANES

**DOI:** 10.3389/fmed.2024.1507100

**Published:** 2024-12-09

**Authors:** Xiao-Ming Zhu, Yan Xu, Jie Zhang

**Affiliations:** Department of Cardiology, Xishan People's Hospital of Wuxi City, Wuxi, China

**Keywords:** heart failure, Cardiometabolic Index, NHANES, prevention strategies, lipids

## Abstract

**Introduction:**

Heart failure is a complex syndrome characterized by impaired cardiac function. Despite improvements in treatment, the prevalence of heart failure continues to rise. The Cardiometabolic Index (CMI), a novel measure combining abdominal obesity and lipid levels, has emerged as a potential predictor of cardiac metabolic risk.

**Methods:**

We analyzed data from the National Health and Nutrition Examination Survey (NHANES) involving 22,586 participants to investigate the association between CMI and heart failure. Multivariable logistic regression models and RCS analysis were used to explore the association between heart failure and CMI after adjusting for potential confounders. Subgroup analyses were performed among populations with different demographic and clinical characteristics.

**Results:**

Our results revealed a significant positive correlation between CMI and heart failure, with odds ratios of 2.77 and 1.87 for the highest quartile after adjusting for confounders. Subgroup analyses indicated heightened risks among older adults and those with hypertension or diabetes. ROC curve analysis demonstrated that CMI offers good diagnostic value for heart failure, surpassing traditional measures like BMI.

**Discussion:**

Our findings suggest that CMI is a valuable tool for assessing the risk of heart failure, particularly in individuals with increased abdominal obesity or abnormal lipid profiles. This highlights the importance of addressing cardiac metabolic health in both prevention and treatment strategies for heart failure. Future research should focus on exploring causal relationships and refining predictive models that incorporate CMI to enhance early detection and intervention.

## Introduction

Heart failure refers to a syndrome caused by various factors leading to impaired cardiac pumping function, where cardiac output fails to meet the basic metabolic needs of tissues, primarily manifesting as dyspnea, limited activity, and fluid retention (1–3). Heart failure can be categorized based on the affected area into left heart failure, right heart failure, and congestive heart failure (4, 5). Although the incidence of heart failure in China has stabilized or declined over the years, its prevalence continues to rise due to aging populations, increased risk factors, and improved effectiveness and survival rates of new therapies (6). Patients with heart failure often experience multiple complications that, if not treated promptly, can create a vicious cycle with heart failure (7). Treatment methods for heart failure include medication, cardiac resynchronization therapy (CRT), and implantable cardioverter-defibrillators (ICD) (8). Over the past few decades, there have been significant breakthroughs in heart failure treatment; however, traditional approaches still face many challenges (9). The mortality rate among heart failure patients remains high, indicating considerable room for improvement in overall treatment (10, 11).

The Cardiometabolic Index (CMI) is a novel obesity index that comprehensively reflects abdominal obesity and lipid levels (12). Cardiac metabolic risk factors play a crucial role in the pathogenesis of heart diseases, forming the theoretical basis for cardiac metabolic risk (13). Overweight or obese individuals, particularly those with abdominal obesity, have a high risk of metabolic abnormalities, making a systematic assessment of cardiac metabolism essential (14). The CMI is a diabetes risk indicator based on triglycerides, HDL-C, height, and waist circumference, useful for evaluating cardiovascular diseases associated with abnormal body fat distribution (12). This index can also assess the risk of hypertension and hyperuricemia related to abnormal body fat distribution and is associated with cardiovascular events and ischemic stroke (15). Guo et al. conducted a large-scale cross-sectional study based on the NHANES database in the U.S. population. The researchers found a positive correlation between CMI and the risk of chronic kidney disease, which may play a key role in the prevention and treatment of this condition (16).

Alterations in energy metabolism are an important characteristic of heart failure, and optimizing myocardial energy metabolism is one of the key strategies for treating heart failure (17, 18). However, the relationship between CMI and heart failure remains unclear. Therefore, we conducted a similar cross-sectional study based on the NHANES database to investigate the correlation between CMI and heart failure.

## Materials and methods

### Study population

**Figure 1:**
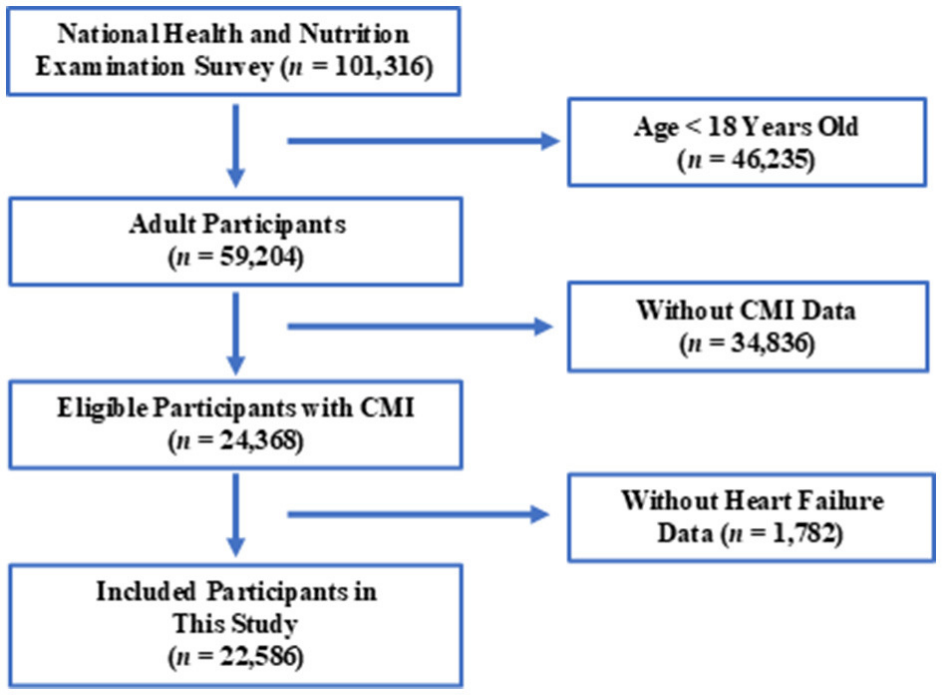
Flowchart of study population enrollment.

The NHANES database, managed by CDC, is the largest population-based national nutrition and health survey globally. Detailed information about the NHANES database can be found on their website: NHANES, accessed on October 4, 2024. This survey has been conducted biennially since 1999 to assess the health and nutritional status of U.S. residents, selecting a representative population. In this study, we included data from 10 consecutive cycles of NHANES, spanning from 1999/2000 to 2017/2018. Inclusion criteria are illustrated in Figure 1.

### Calculation of CMI

Cardiac Metabolic Index (CMI), derived from anthropometric measurements and blood samples. Blood samples were typically collected in the survey vehicles or designated locations. Using these measurements, the waist-to-height ratio (WHtR) and CMI were calculated as follows:


$$\begin{array}{l} \text{WHtR}=\text{Waist circumference (cm)/Height (cm),}\\ \text{CMI}=\text{Triglycerides (mmol/L)/High-Density Lipoprotein}\\ \text{Cholesterol (HDL-C, mmol/L)}\times \text{WHtR}. \end{array}$$

WC was measured at the midpoint between the lowest rib and the iliac crest by experienced staff of NHANES. BMI was calculated using participants' weight and height measurements, where BMI = weight (kg)/height (m^2^). Height was measured using a calibrated digital scale, and height was recorded using a stadiometer with participants in a standing position, without shoes. Serum lipid-related indicators levels were measured using standard enzymatic methods. Blood samples were collected after an overnight fast (at least 8 h), and the measurements were performed using a certified clinical laboratory.

### Heart failure

Congestive Heart Failure (CHF) was confirmed based on the MCQ questionnaire, which has been validated in prior studies for the effectiveness of self-reported heart failure. Participants were asked, “Has a doctor or other health professional ever told you that you have HF?”

### Covariates

Demographic information was obtained from the demographic questionnaire. Participants provided information on smoking status, alcohol consumption, and medical history through health questionnaires. Blood samples were collected after fasting for at least 8 h to assess biochemical markers. Based on previous research, potential confounding factors affecting heart failure were selected to mitigate their influence on the outcomes. Ultimately, the following covariates were collected and adjusted for: age, sex, education level, ethnicity, BMI, smoking, drinking, diabetes, and hypertension.

### Statistical methods

The analyses were conducted using statistical software R. Continuous variables as means and 95% CI, while categorical variables were reported as percentages and 95% CI. All analyses incorporated appropriate sample weights. This ensured that the estimates were representative of the U.S. population. Chi-square tests were used for categorical variables, while independent *t*-tests were applied for continuous variables. To determine the independent association between CMI and heart failure, multivariable logistic regression analyses were conducted. We selected weighted regression models to account for the complex stratified sampling design of the NHANES data, which includes oversampling of certain population subgroups and differential probabilities of selection. By incorporating weights, we ensure that the estimates are representative of the general population, thereby mitigating the risk of biased results due to the sampling design. The model was adjusted for potential confounders. To assess the potential non-linear relationship between CMI and the risk of heart failure, we employed restricted cubic splines (RCS) in our logistic regression model. We included three knots at predefined percentiles (e.g., the 10th, 50th, and 90th percentiles) of the CMI distribution to model the relationship effectively. To evaluate the diagnostic performance of the CMI in predicting HF, we performed a ROC curve analysis. AUC was calculated to quantify the overall predictive accuracy of CMI for heart failure.

## Results

### Study population

As shown in Table 1, younger individuals (18–40 years) dominate the lowest CMI quartile (Q1) with 47.84%, which decreases progressively across quartiles, reaching 30.39% in the highest quartile (Q4). Conversely, the percentage of individuals aged 60-80 increases from 15.93% in Q1 to 28.61% in Q4. Females are more prevalent in the lower CMI quartiles (58.51% in Q1), while the percentage of males increases across quartiles, reaching 57.06% in Q4. Higher education levels are more common in the lower quartiles (66.58% in Q1), but as the CMI increases, fewer individuals have education above high school (52.08% in Q4). The prevalence of diabetes (DM), fasting blood glucose (FBG), and glycated hemoglobin (HbA1c) all increase across quartiles. In Q4, 29.08% have diabetes, and the average HbA1c is 5.93%. Coronary heart disease (CHD), angina, and heart attacks are significantly more prevalent in higher CMI quartiles. Our study highlights higher CMI is associated with older age, male gender, lower education, higher diabetes rates, obesity, and increased cardiovascular risks? Moreover, the baseline characteristics of participants with and without heart failure were shown in Supplementary Table S1.

**Table 1:** Baseline characteristics of different CMI quartile populations.

**Variables**	**CMI–Q1 (0.02–0.10)**	**CMI–Q2 (0.10–0.14)**	**CMI–Q3 (0.14–0.22)**	**CMI–Q4 (0.22–2.52)**	***P* value**
Age, %					<0.001^***^
18–40 years	47.84 (45.70, 49.99)	41.60 (39.74, 43.46)	35.40 (33.74, 37.05)	30.39 (28.77, 32.01)	
40–60 years	36.22 (34.18, 38.27)	37.51 (35.70, 39.32)	39.74 (38.15, 41.34)	41.00 (39.20, 42.79)	
60–80 years	15.93 (14.48, 17.38)	20.89 (19.39, 22.39)	24.86 (23.28, 26.44)	28.61 (26.90, 30.33)	
Gender, %					<0.001^***^
Female	58.51 (56.83, 60.19)	52.14 (50.54, 53.73)	50.54 (48.91, 52.16)	42.94 (41.41, 44.46)	
Male	41.49 (39.81, 43.17)	47.86 (46.27, 49.46)	49.46 (47.84, 51.09)	57.06 (55.54, 58.59)	
Ethnicity, %					<0.001^***^
Non-Hispanic White	66.77 (64.55, 69.00)	68.52 (66.16, 70.89)	68.42 (65.91, 70.92)	72.28 (69.92, 74.63)	
Non-Hispanic Black	15.34 (13.74, 16.95)	11.57 (10.22, 12.91)	8.85 (7.76, 9.94)	5.45 (4.71, 6.20)	
Mexican American	5.55 (4.72, 6.37)	7.91 (6.74, 9.09)	9.72 (8.34, 11.09)	10.21 (8.79, 11.62)	
Other Hispanic	4.87 (3.93, 5.82)	5.58 (4.51, 6.64)	6.42 (5.13, 7.70)	5.68 (4.51, 6.85)	
Others	7.46 (6.46, 8.46)	6.42 (5.55, 7.29)	6.60 (5.72, 7.48)	6.38 (5.45, 7.32)	
Education, %					<0.001^***^
Below high school	3.70 (3.09, 4.31)	5.32 (4.67, 5.98)	7.79 (6.94, 8.64)	7.88 (7.04, 8.71)	
High school	29.72 (27.80, 31.63)	34.94 (32.97, 36.91)	37.54 (35.64, 39.45)	40.04 (37.96, 42.12)	
Above high school	66.58 (64.42, 68.74)	59.74 (57.70, 61.77)	54.67 (52.53, 56.80)	52.08 (49.98, 54.19)	
DM, %	4.48 (3.82, 5.13)	8.71 (7.84, 9.57)	15.94 (14.69, 17.19)	29.08 (27.38, 30.79)	<0.001^***^
FBG, mmol/L	5.36 (5.33, 5.39)	5.60 (5.56, 5.63)	5.90 (5.85, 5.96)	6.55 (6.46, 6.63)	<0.001^***^
HBA1c, %	5.32 (5.30, 5.34)	5.46 (5.43, 5.48)	5.62 (5.60, 5.65)	5.93 (5.88, 5.98)	<0.001^***^
Smoking, %	19.10 (17.64, 20.56)	21.25 (19.59, 22.91)	23.00 (21.49, 24.51)	22.40 (21.01, 23.79)	<0.001^***^
Drinking, %	89.73 (88.50, 90.95)	88.87 (87.61, 90.14)	89.23 (87.98, 90.49)	88.06 (86.70, 89.42)	0.12
BMI, %					<0.001^***^
Normal weight	60.03 (58.42, 61.65)	35.63 (33.73, 37.53)	18.36 (16.95, 19.76)	8.69 (7.74, 9.64)	
Obesity	11.31 (10.32, 12.29)	26.56 (24.98, 28.14)	44.32 (42.45, 46.20)	60.59 (59.04, 62.13)	
Over weight	28.66 (27.14, 30.17)	37.81 (36.14, 39.48)	37.32 (35.59, 39.04)	30.73 (29.23, 32.22)	
Hypertension, %	22.88 (21.24, 24.53)	32.83 (31.08, 34.57)	42.68 (40.93, 44.44)	54.03 (52.05, 56.00)	<0.001^***^
SBP, mmHg	116.91 (116.33, 117.49)	120.48 (119.90, 121.07)	123.28 (122.63, 123.93)	125.66 (125.14, 126.18)	<0.001^***^
DBP, mmHg	68.85 (68.42, 69.28)	70.20 (69.76, 70.65)	71.24 (70.77, 71.72)	72.12 (71.66, 72.58)	<0.001^***^
CHD, %	1.34 (0.99, 1.68)	2.75 (2.17, 3.33)	3.58 (2.99, 4.16)	6.36 (5.52, 7.20)	<0.001^***^
Angina, %	0.85 (0.58, 1.11)	1.72 (1.26, 2.17)	2.58 (2.08, 3.07)	4.47 (3.77, 5.16)	<0.001^***^
Heart attack, %	1.31 (0.95, 1.66)	2.85 (2.36, 3.34)	3.68 (2.97, 4.38)	6.09 (5.34, 6.85)	<0.001^***^

BMI, body mass index; DM, diabetes; FBG, fasting blood glucose; HbA1c, glycated hemoglobin; HF, heart failure. ^***^P < 0.001.

### Association of CMI and heart failure

**Figure 2:**
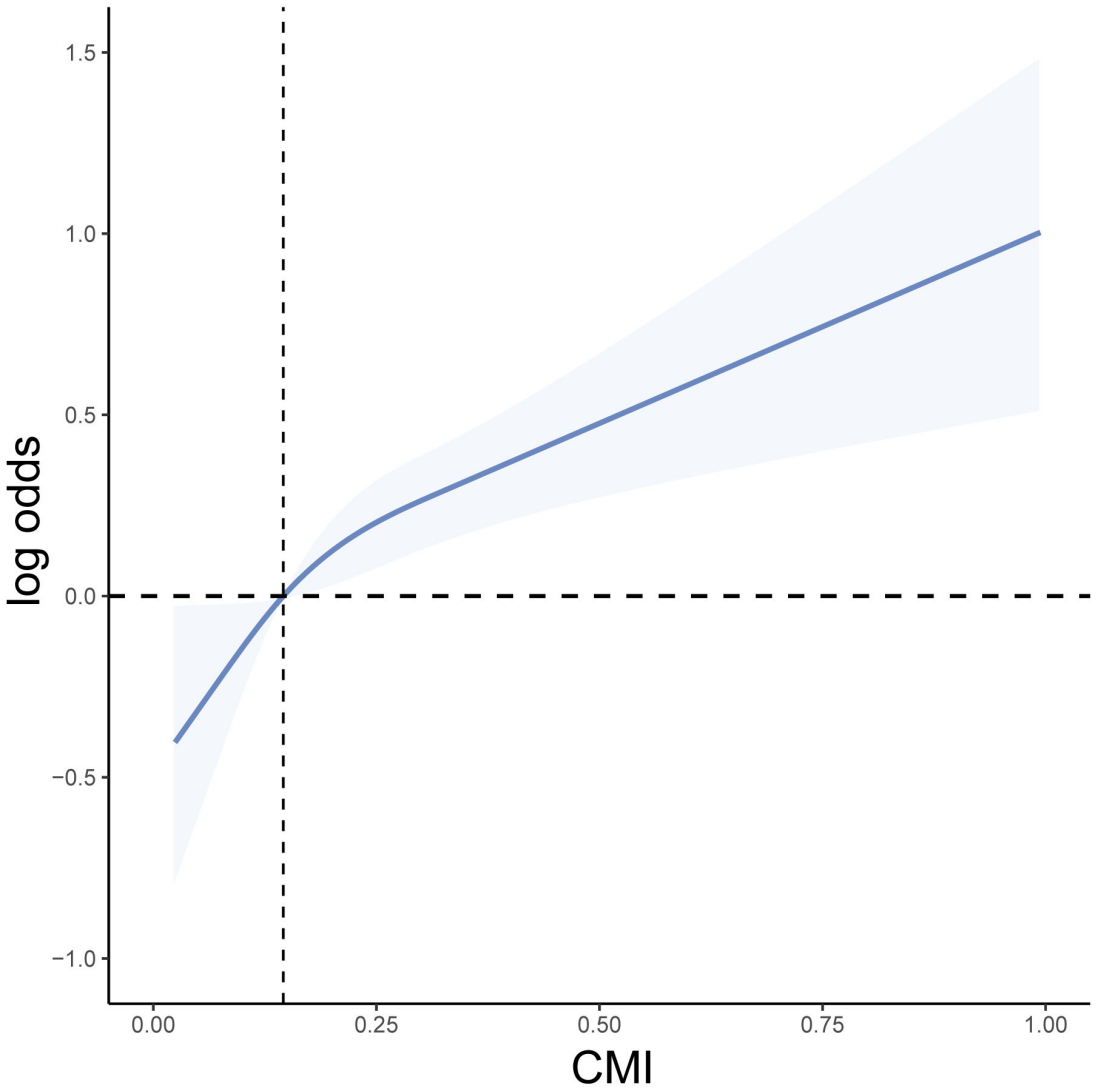
RCS curve of the association between CMI and heart failure.

Weighted logistic regression results showed that as CMI increased, the risk of heart failure significantly elevated. In the unadjusted model, compared to the lowest quartile (Q1), the odds ratio (OR) for the second quartile (Q2) was 2.03 (95% CI: 1.39–2.96, *P* < 0.001), for the third quartile (Q3) was 2.82 (95% CI: 1.95–4.09, *P* < 0.001), and for the highest quartile (Q4) was 5.38 (95% CI: 3.75–7.72, *P* < 0.001). In Model I, adjusted for age, sex, and race/ethnicity, the OR for Q4 remained 2.77 (95% CI: 1.92–3.99, *P* < 0.001). After further adjusting for BMI, education level, smoking, drinking, diabetes, and hypertension in Model II, the OR for Q4 was 1.87 (95% CI: 1.26–2.76, *P* = 0.002). Therefore, CMI is significantly associated with the occurrence of heart failure (Table 2). Additionally, we used RCS curve for a visual analysis of the relationship between CMI and the risk of heart failure. We found that as CMI levels increased, the risk of heart failure also continuously increased, showing a linear positive correlation between the two (Figure 2). The NHANES data has a complex multistage sampling design, and we use weighted regression models in the primary analysis to ensure that the sample represents the general population. Unweighted logistic regression can help further test the robustness of the model, verify the necessity of the weighted design, and reveal potential sampling bias. Therefore, we applied unweighted analysis as a sensitivity analysis to verify whether the results remain consistent without considering the weighting. In sensitivity analysis, after adjusting for confounders, CMI was still significantly associated with HF in Q4 (OR 1.69, 95% CI 1.28–2.26, *P* < 0.001). These findings in sensitive analysis suggest consists with Amin analysis (Table 3).

**Table 2:** Weighted logistic regression analysis of CMI and HF.

**Cardiometabolic Index**	**Non-adjusted model**		**Model I**		**Model II**	
	**OR [95% CI]**	***P*** **value**	**OR [95% CI]**	***P*** **value**	**OR [95% CI]**	***P*** **value**
Q1	Reference	-	Reference	-	Reference	-
Q2	2.03 (1.39, 2.96)	<0.001^***^	1.54 (1.04, 2.28)	0.03^*^	1.25 (0.82, 1.90)	0.29
Q3	2.82 (1.95, 4.09)	<0.001^***^	1.67 (1.14, 2.44)	0.01^*^	1.25 (0.84, 1.87)	0.27
Q4	5.38 (3.75, 7.72)	<0.001^***^	2.77 (1.92, 3.99)	<0.001^***^	1.87 (1.26, 2.76)	0.002^**^

Model I adjusted for age, sex, and race/ethnicity. Model II adjusted for age, sex, education level, ethnicity, BMI, smoking, drinking, diabetes, hypertension. CMI, Cardiometabolic Index; HF, heart failure; BMI, family income.

^*^P < 0.05, ^**^P < 0.01, ^***^P < 0.001.

**Table 3:** Unweighted logistic regression analysis of CMI and HF.

**Cardiometabolic Index**	**Non-adjusted model**		**Model I**		**Model II**	
	**OR [95% CI]**	***P*** **value**	**OR [95% CI]**	***P*** **value**	**OR [95% CI]**	***P*** **value**
Q1	Reference	-	Reference	-	Reference	-
Q2	1.88 (1.41, 2.53)	<0.001^***^	1.29 (0.97, 1.73)	0.08^*^	1.11 (0.82, 1.50)	0.51
Q3	2.76 (2.11, 3.66)	<0.001^***^	1.55 (1.18, 2.06)	0.002^**^	1.32 (1.00, 1.77)	0.05^*^
Q4	4.61 (3.57, 6.02)	<0.001^***^	2.25 (1.73, 2.97)	<0.001^***^	1.69 (1.28, 2.26)	<0.001^***^

Model I adjusted for age, sex, and race/ethnicity. Model II adjusted for age, sex, education level, ethnicity, BMI, smoking, drinking, diabetes, hypertension.

CMI, Cardiometabolic Index; HF, heart failure; BMI, family income.

^*^P < 0.05, ^**^P < 0.01, ^***^P < 0.001.

### Subgroup analysis

**Figure 3:**
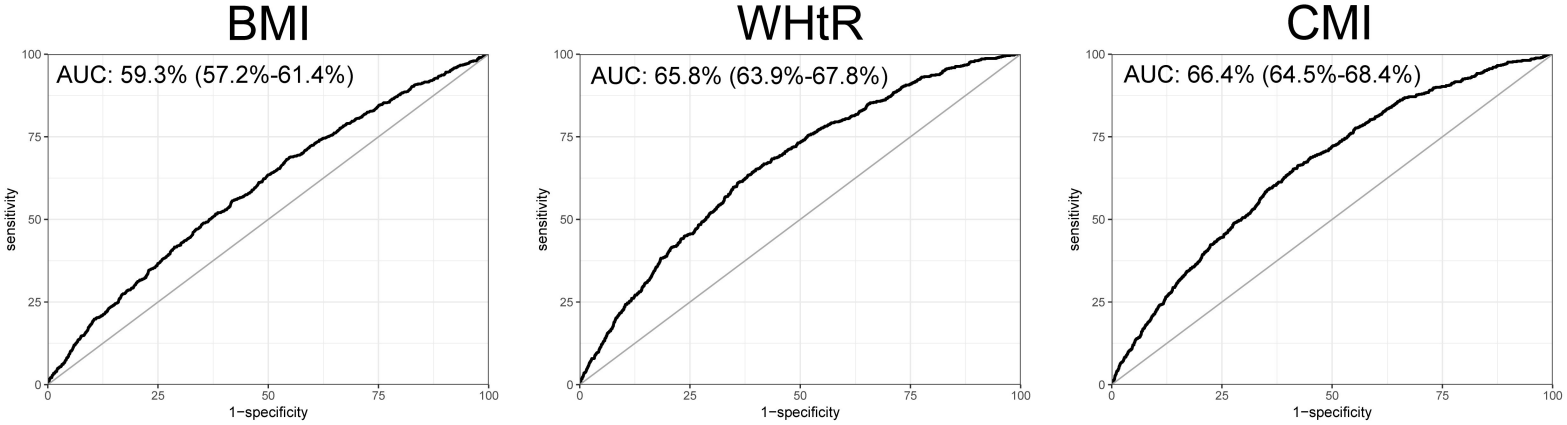
ROC curves of BMI, WtHR, and CMI for predicting heart failure.

In addition, we conducted a detailed subgroup analysis to explore the association between CMI and HF in populations with different demographic characteristics. In the 40–60 and 60–80 age groups, higher quartiles of Cardiometabolic Index (Q3 and Q4) were significantly associated with the risk of heart failure, particularly in the 60–80 age group where the risk was highest in Q4 (OR = 3.52, 95% CI: 2.22–5.58, *p* < 0.0001). Among both females and males, higher quartiles of Cardiometabolic Index (Q3 and Q4) were significantly associated with heart failure risk, with an OR of 3.26 (95% CI: 1.97–5.40, *p* < 0.0001) for females in Q4 and an OR of 3.35 (95% CI: 1.97–5.71, *p* < 0.0001) for males in Q4. In individuals with hypertension, higher quartiles of Cardiometabolic Index (Q2 to Q4) were all significantly associated with the risk of heart failure, especially in Q4 (OR = 2.81, 95% CI: 1.93–4.08, *p* < 0.0001), while in individuals without hypertension, only Q4 showed a significant association (OR = 3.17, 95% CI: 1.47–6.83, *p* = 0.003). For individuals without diabetes, higher quartiles of Cardiometabolic Index (Q3 and Q4) were significantly associated with heart failure risk. In individuals with diabetes, only Q4 significantly increased the risk of heart failure (OR = 1.79, 95% CI: 1.05–3.08, *p* = 0.03). The association between CMI and heart failure was positively correlated across different age groups, hypertension, and diabetes status. Particularly, individuals who are older or have hypertension or diabetes exhibited a significantly higher risk in higher quartiles of Cardiometabolic Index (Figure 3).

### ROC curve

**Figure 4:**
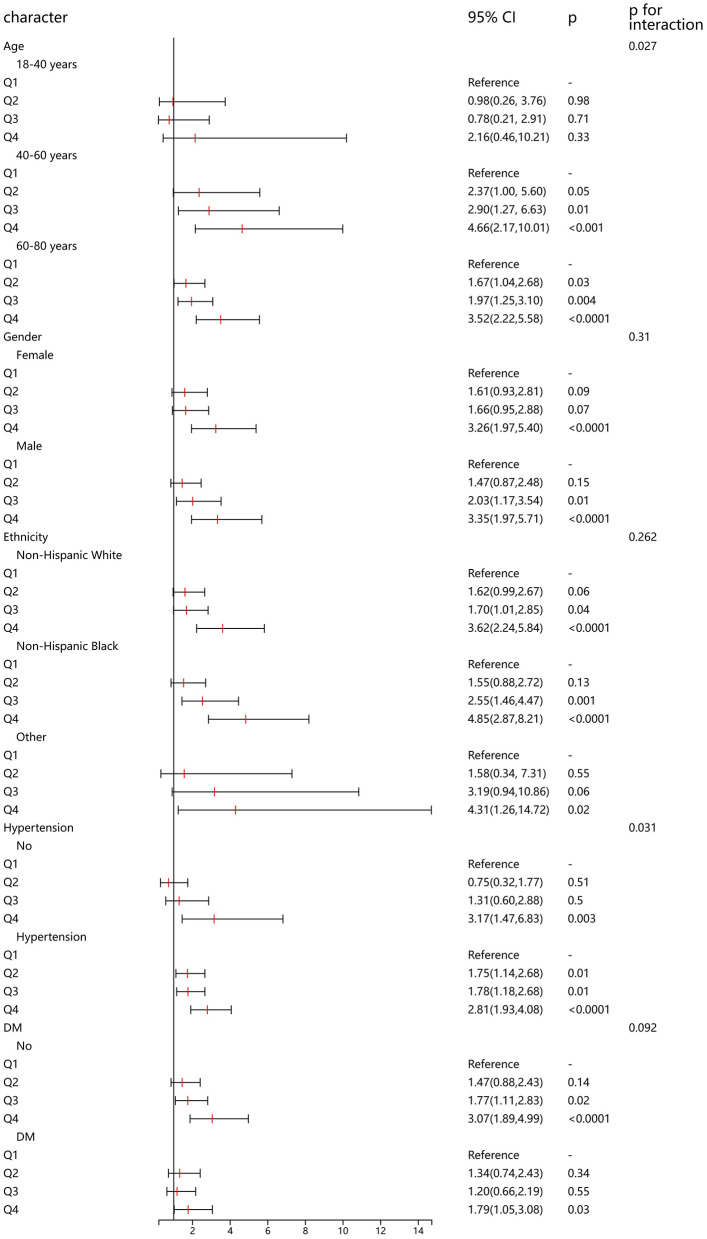
Subgroup analysis of the association between CMI and heart failure.

To further explore the efficacy of CMI in the diagnosis and prediction of heart failure, we plotted the ROC curves for CMI, BMI, and WHtR. We found that the ROC curve of CMI demonstrated good diagnostic efficacy, with an AUC of 66.4% (95% confidence interval: 64.5% to 68.4%). The AUC for CMI was greater than that of BMI and WHtR in terms of their diagnostic efficacy for heart failure (Figure 4).

## Discussion

A large body of research has demonstrated that cardiac metabolic activity is crucial for heart failure (19, 20). We conducted a large-scale cross-sectional analysis based on the NHANES database, revealing a positive correlation between CMI and heart failure. Clinical practitioners should pay more attention to cardiac metabolism.

CMI is a measure used to assess cardiac metabolic function (21), to provide a more comprehensive reflection of heart metabolic health (22). Choi et al. firstly revealed CMI in a 2017 study as a potential clinical indicator of lipid metabolism abnormalities, suggesting it may hold significant value in predicting renal outcomes (23). Wang et al. found that CMI levels are closely related to endometriosis, with the correlation strengthening. By regularly monitoring CMI levels, doctors may be able to identify women at risk for endometriosis earlier (13). Furthermore, studies have shown a significant positive correlation between CMI and the risk of gestational diabetes mellitus, and higher CMI levels can predict the occurrence of gestational diabetes mellitus during pregnancy (24). The mechanisms of myocardial energy metabolism disorders primarily include impaired energy generation and utilization (25). Numerous studies have shown that obesity can lead to both energy generation and utilization impairments (26, 27). The myocardium primarily derives energy through the aerobic oxidation of various substrates, including fatty acids and glucose (28, 29). ATP generated during oxidative phosphorylation in myocardial cells is hydrolyzed by the myosin head ATPase during the excitation-contraction coupling process, providing energy for myocardial contraction (27, 30, 31). Research has found that when myocardial overload leads to hypertrophy, structural changes occur in myocardial contractile proteins, resulting in decreased activity of myosin head ATPase and impaired ATP hydrolysis (32). This disruption in energy utilization subsequently weakens myocardial contractility (33). Therefore, the metabolic activities of the heart are closely related to heart failure. Based on the results of present study, CMI could serve as a practical tool for identifying individuals at high risk of heart failure (HF) before the onset of clinical symptoms. It also has the potential to be utilized to categorize patients based on their cardiometabolic risk, allowing for tailored clinical management.

There is distinguishing between different types of heart failure, such as heart failure with preserved ejection fraction (HFpEF) and heart failure with reduced ejection fraction (HFrEF), is an important aspect of understanding the clinical utility of the CMI. However, due to the limitations of the available data in the NHANES dataset, we were unable to obtain detailed information regarding the specific type of heart failure for each individual participant. As such, we could not analyze the impact of CMI on different heart failure subtypes in this study. Heart failure poses significant dangers, leading to reduced activity levels, general fatigue, and even impacting daily life and work (34). Additionally, heart failure can result in decreased cardiac pumping function, which, in severe cases, may lead to shock or cardiac arrest (35). The pathophysiological changes associated with heart failure are multifaceted, primarily involving pathophysiological changes, ventricular remodeling, activation of neuroendocrine and sympathetic nervous systems, and hemodynamic abnormalities (36, 37). The mechanisms underlying heart failure are complex (38). Although different primary heart diseases lead to varying pathophysiological mechanisms of heart failure, it is currently believed that abnormalities in myocardial energy metabolism and energy utilization impairment represent common pathways contributing to heart failure (39, 40). Increasing attention is being paid to the remodeling of myocardial energy metabolism alongside structural and electrical remodeling of the heart caused by heart failure (41, 42). Patients with heart failure exhibit energy metabolism abnormalities, it can lead to ventricular remodeling, resulting in impaired cardiac contractile and/or diastolic function (43, 44). Improving myocardial energy metabolism can enhance the prognosis of heart failure patients, offering new avenues for treatment. Several studies have identified a relationship between various factors, such as metabolic disorders, and heart failure (45, 46). Of note, we found that the association of CMI with HF was more pronounced among elderly population, we believe that this was attributed to higher risk of occurrence of HF among aging participants. With aging, there may be alterations in neurohormonal regulation, including increased sympathetic nervous system activity and elevated levels of natriuretic peptides, which may exacerbate the development of heart failure in predisposed individuals. Moreover, the age-related cardio-injury and vascular dysfunction are also related to higher risk of HF among elderly.

Our study has several limitations that need to be emphasized. Firstly, the onset and progression of heart failure are often related to many factors, making it difficult to predict disease incidence based solely on changes in a single indicator. While we accounted for key comorbidities, there may be other potential confounding factors that were not included in the analysis, such as specific inflammatory markers and genetic predispositions, due to data availability constraints in the NHANES dataset. Secondly, cross-sectional studies cannot be used to infer causal relationships, and the conclusions of this study may require further confirmation through additional prospective research. Furthermore, we cannot ensure that there is no reporting bias in the population of the database, nor can we accurately adjust for all confounding factors. It is important to note that previous studies, including many large-scale epidemiological studies, have successfully relied on self-reported data for HF diagnosis, particularly when more objective clinical data were not available (47, 48). The National Health and Nutrition Examination Survey (NHANES), from which our data were derived, is widely recognized for its rigorous methodology and high-quality data collection standards. The survey uses validated questions and standardized procedures to ensure consistency and reliability in the information gathered. Therefore, despite the self-report nature of the HF diagnosis, we believe the NHANES data is of high quality and contributes meaningfully to our analysis. There are certain differences in health indicators between the American population and the other population, so we cannot reasonably extrapolate our findings to the other population. Moreover, we did not establish a predictive model incorporating CMI in this study, therefore, future research should focus more on this aspect, while CMI demonstrates some potential in diagnosing heart failure, its diagnostic performance may still require improvement before it can be considered a robust standalone tool in clinical practice. Besides, as this study is based on cross-sectional NHANES data, the information available on heart failure severity and subtypes is limited. The strengths of our study is its exploration of the positive correlation between CMI and heart failure for the first time, using a relatively large sample size. Additionally, we employed weighted sampling analysis to further investigate among different populations.

## Conclusion

In this cross-sectional analysis based on the NHANES, including 22,586 participants, using weighted analysis methods, we found a positive correlation between CMI and heart failure. Our findings suggest that CMI may serve as an effective tool for early identification and risk stratification in heart failure, particularly in populations at risk due to metabolic abnormalities. This contributes to the growing body of evidence linking metabolic dysfunction to cardiovascular outcomes, with implications for preventive healthcare strategies. However, more prospective studies still needed.

## Data Availability

The original contributions presented in the study are included in the article/Supplementary material, further inquiries can be directed to the corresponding author.
