# Supplementary material for: Cardiometabolic Index is associated with heart failure: a cross-sectional study based on NHANES
Source: Front Med (Lausanne). 2024 Dec 9;11:1507100. doi: 10.3389/fmed.2024.1507100 (PMC11663657; doi:10.3389/fmed.2024.1507100)
Supplement: Supplementary file 1 [file Table_1.docx]

**Table S1. Baseline Characteristics among Non-HF and HF group.**

| Variables | Overall  (n = 22,586) | Non-HF  (n = 21,878) | HF  (n = 708) | *P* value |
| --- | --- | --- | --- | --- |
| Age, % |  |  |  | <0.001*** |
| 18-40 years | 38.68 (37.02, 40.35) | 39.91 (38.73, 41.09) | 5.68 (3.34, 8.03) |  |
| 40-60 years | 38.08 (36.28, 39.89) | 38.77 (37.75, 39.79) | 27.88 (23.21, 32.55) |  |
| 60-80 years | 22.09 (20.67, 23.51) | 21.32 (20.31, 22.33) | 66.43 (61.34, 71.53) |  |
| Gender, % |  |  |  | 0.06 |
| Female | 51.27 (49.28, 53.25) | 51.39 (50.70, 52.08) | 46.31 (41.29, 51.34) |  |
| Male | 48.73 (46.71, 50.75) | 48.61 (47.92, 49.30) | 53.69 (48.66, 58.71) |  |
| Ethnicity, % |  |  |  | 0.002** |
| Non-Hispanic White | 68.92 (64.67, 73.17) | 68.81 (66.79, 70.82) | 73.74 (69.95, 77.53) |  |
| Non-Hispanic Black | 10.47 (9.54, 11.40) | 10.42 (9.34, 11.50) | 12.61 (9.95, 15.28) |  |
| Mexican American | 8.26 (7.30, 9.22) | 8.36 (7.29, 9.43) | 4.09 (2.52, 5.66) |  |
| Other Hispanic | 5.61 (4.68, 6.55) | 5.64 (4.70, 6.59) | 4.38 (2.36, 6.39) |  |
| Others | 6.73 (6.08, 7.39) | 6.77 (6.13, 7.42) | 5.19 (2.70, 7.67) |  |
| Education, % |  |  |  | <0.001*** |
| Below high school | 6.08 (5.59, 6.57) | 5.90 (5.41, 6.39) | 13.78 (11.05, 16.50) |  |
| High school | 35.35 (33.35, 37.35) | 35.09 (33.75, 36.43) | 47.44 (42.99, 51.89) |  |
| Above high school | 58.49 (55.87, 61.11) | 59.01 (57.51, 60.51) | 38.78 (33.77, 43.80) |  |
| DM, % | 14.16 (13.29, 15.03) | 13.40 (12.73, 14.08) | 45.19 (40.77, 49.62) | <0.001*** |
| FBG, mmol/L | 5.83 (5.80, 5.87) | 5.81 (5.78, 5.84) | 6.84 (6.61, 7.07) | <0.001*** |
| HBA1c, % | 5.57 (5.55, 5.59) | 5.56 (5.54, 5.58) | 6.22 (6.10, 6.35) | <0.001*** |
| Smoking, % | 21.35 (20.06, 22.65) | 21.35 (20.34, 22.36) | 21.97 (17.31, 26.64) | 0.79 |
| Drinking, % | 82.58 (79.18, 85.99) | 89.07 (88.11, 90.03) | 85.79 (82.18, 89.40) | 0.05* |
| BMI, % |  |  |  | <0.001*** |
| Normal weight | 31.58 (30.05, 33.12) | 31.90 (30.90, 32.89) | 20.04 (16.26, 23.81) |  |
| Obesity | 34.79 (33.07, 36.51) | 34.50 (33.53, 35.47) | 48.40 (43.51, 53.30) |  |
| Over weight | 33.52 (32.07, 34.97) | 33.61 (32.75, 34.46) | 31.56 (27.44, 35.67) |  |
| Hypertension, % | 37.57 (35.71, 39.44) | 36.50 (35.45, 37.56) | 81.65 (77.65, 85.65) | <0.001*** |
| SBP, mmHg | 121.42 (121.05, 121.80) | 121.25 (120.88, 121.62) | 128.66 (126.70, 130.61) | <0.001*** |
| DBP, mmHg | 70.54 (70.22, 70.87) | 70.63 (70.31, 70.95) | 67.05 (65.78, 68.32) | <0.001*** |
| CHD, % | 3.42 (3.04, 3.80) | 2.60 (2.31, 2.89) | 38.60 (33.62, 43.58) | <0.001*** |
| Angina, % | 2.34 (2.04, 2.64) | 1.78 (1.55, 2.00) | 26.11 (21.73, 30.50) | <0.001*** |
| Heart attack, % | 3.40 (3.03, 3.78) | 2.43 (2.16, 2.69) | 43.90 (39.19, 48.60) | <0.001*** |

Abbreviations: BMI, body mass index; DM, diabetes; FBG, fasting blood glucose; HbA1c, glycated hemoglobin; HF, heart failure. * *P* value<0.05, ** *P* value<0.01, *** *P* value<0.001.
